# Multiparametric imaging hippocampal neurodegeneration and functional connectivity with simultaneous PET/MRI in Alzheimer’s disease

**DOI:** 10.1007/s00259-020-04752-8

**Published:** 2020-03-10

**Authors:** Shaozhen Yan, Chaojie Zheng, Bixiao Cui, Zhigang Qi, Zhilian Zhao, Yanhong An, Liyan Qiao, Ying Han, Yun Zhou, Jie Lu

**Affiliations:** 1 Department of Radiology, Xuanwu Hospital, Capital Medical University, Beijing, China; 2 Mallinckrodt Institute of Radiology, Washington University School of Medicine, 510 Kingshighway Blvd., St. Louis, MO USA; 3 Beijing Key Laboratory of Magnetic Resonance Imaging and Brain Informatics, Beijing, China; 4 Department of Nuclear Medicine, Xuanwu Hospital, Capital Medical University, Beijing, China; 5 Department of Neurology, Yuquan Hospital, Clinical Neuroscience Institute, Medical Center, Tsinghua University, Beijing, China; 6 Department of Neurology, Xuanwu Hospital, Capital Medical University, Beijing, China

**Keywords:** Alzheimer’s disease, Hippocampal subregions, Neurodegeneration, Hybrid PET/MRI, Voxel-based morphometric analysis

## Abstract

**Purpose:**

The objective of this study is to investigate the hippocampal neurodegeneration and its associated aberrant functions in mild cognitive impairment (MCI) and Alzheimer’s disease (AD) patients using simultaneous PET/MRI.

**Methods:**

Forty-two cognitively normal controls (NC), 38 MCI, and 22 AD patients were enrolled in this study. All subjects underwent ^18^F-FDG PET/functional MRI (fMRI) and high-resolution T1-weighted MRI scans on a hybrid GE Signa PET/MRI scanner. Neurodegeneration in hippocampus and its subregions was quantified by regional gray matter volume and ^18^F-FDG standardized uptake value ratio (SUVR) relative to cerebellum. An iterative reblurred Van Cittert iteration method was used for voxelwise partial volume correction on ^18^F-FDG PET images. Regional gray matter volume was estimated from voxel-based morphometric analysis with MRI. fMRI data were analyzed after slice time correction and head motion correction using statistical parametric mapping (SPM12) with DPARSF toolbox. The regions of interest including hippocampus, cornu ammonis (CA1), CA2/3/dentate gyrus (DG), and subiculum were defined in the standard MNI space.

**Results:**

Patient groups had reduced SUVR, gray matter volume, and functional connectivity compared to NC in CA1, CA2/3/DG, and subiculum (AD < MCI < NC). There was a linear correlation between the left CA2/3DG gray matter volume and ^18^F-FDG SUVR in AD patients (*P* < 0.001, *r* = 0.737). Significant correlation was also found between left CA2/3/DG-superior medial frontal gyrus functional connectivity and left CA2/3/DG hypometabolism in patients with AD. The functional connectivity of right CA1-precuneus in patients with MCI and right subiculum-superior frontal gyrus in patients with AD was positively correlated with mini mental status examination scores (*P* < 0.05).

**Conclusion:**

Our findings demonstrate that the associations existed at subregional hippocampal level between the functional connectivity measured by fMRI and neurodegeneration measured by structural MRI and ^18^F-FDG PET. Our results may provide a basis for precision neuroimaging of hippocampus in AD.

## Introduction

The hippocampus, as a structure playing a key role in cognitive processes, is known to remain central to the understanding of the Alzheimer’s disease (AD) pathophysiology with sensitivity to the neurofibrillary tangle development and a strong association with progression to AD [1–3]. It is widely recognized that the hippocampus is heterogeneous and can be divided into subregions with different functions and vulnerabilities to neurodegenerative diseases [4–6]. While the hippocampal subregions are thought to exhibit distinct functions, the neural substrate for aberrant functionalities remains elusive.

Several imaging studies have identified and investigated hippocampal subregions with functional MRI (fMRI) in healthy young and aging human brain [7, 8]. Using in vivo MRI, the hippocampus can be divided into three subregions: cornu ammonis (CA1), CA2/3/dentate gyrus (CA2/3/DG), and subiculum [9]. Resting-state fMRI studies showed that the disrupted total hippocampal connectivity [10], right CA1 and left CA2 subregions connectivity [11], and subiculum network (functional connectivity with frontal and posterior cingulate cortex [PCC] regions) [12] in mild cognitive impairment (MCI) and AD patients were strongly associated with memory impairment [5, 13, 14]. In addition to fMRI, the fluorine 18 (^18^F) fluorodeoxyglucose (^18^F-FDG) PET showed that the glucose metabolism of the left hippocampal body CA2/3 and CA4/DG subregions was significantly lower in the early AD group than in the control group [15]. In MCI patients, we found that the left hippocampal CA2 functional connectivity measured by resting-state fMRI was associated with decreased dorsal raphe nuclei binding potential measured by [^11^C]DASB PET [16].

The previous structural MRI, fMRI, and ^18^F-FDG PET studies have reported reduced volume, disrupted intrinsic activity, and hypometabolism of hippocampus and hippocampal subregions in both AD and MCI patients [11, 12, 15, 17, 18]. However, there is still a lack of systematic examinations of the relationship between AD pathology in the hippocampal subregions and cognitive performance. More importantly, few have related intrinsic activity and metabolism based on the detailed subregional analyses on hippocampal. While hippocampus is widely recognized for its subregions with distinct functions [6], the localization of pathologies would indicate the specific roles of hippocampal subregions in symptomatology. Examining the relationship between intrinsic activity and metabolism within hippocampal subregions may also provide insight into disease pathogenesis.

Hybrid PET/MRI simultaneously evaluates resting-state brain structure, intrinsic activity, and glucose metabolism, which would provide optimal spatial and temporal registration of both modalities and clarify how neuronal function is impaired and contributes to the mechanisms underlying AD [19]. We hypothesized that different subregions have various contributions to the functionalities of hippocampus, and we aimed to investigate the aberrant of hippocampal subregions in MCI and AD patients regarding functional connectivity and metabolism using hybrid PET/MRI.

## Methods

### Participants

A total of 102 right-handed subjects were included in this study, comprising of 42 normal controls (NC), 38 MCI, and 22 AD participants. Ethical approval was obtained from the Medical Research Ethics Committee of Xuanwu Hospital, Capital Medical University. Written informed consent was obtained from each participant and/or their legal representative before the PET/MR scan. Clinical diagnosis was established on the basis of a standard dementia screening that included medical history review, physical and neurological examinations, laboratory tests, neuropsychological tests, and brain ^18^F-FDG PET/MRI. Criteria for selecting the patients with AD met the National Institute of Neurological and Communicative Diseases and Stroke/Alzheimer’s Disease and Related Disorders Association criteria for probable AD [20, 21]. MCI was diagnosed according to the criteria of Petersen and colleagues [22]. All participants were assessed by neurologist with expertise in AD disorders. We excluded participants with Fazekas scores > 2 to minimize the effect of vascular factors on functional connectivity [23], and other neurologic, psychiatric, or brain parenchyma diseases (e.g., stroke, tumors, and trauma) potentially related to cognitive impairment. The interval between neuropsychological assessments and simultaneous PET/MRI scan was within 30 days. To estimate cerebral microvascular impairment, the Fazekas scores were assessed by two experienced neuroradiologists [24].

### PET/MRI data acquisition

Imaging data were collected with an integrated simultaneous time-of-flight (ToF) PET/MRI (Signa PET/MRI, GE Healthcare, WI, USA) by using a 19-channel head and neck union coil. Participants were scanned under resting conditions with eyes closed and dimmed light. The PET/MRI acquisition protocol was as same as our previous studies [25, 26]. A sagittal 3D brain in volume (3D BRAVO) T1-weighted sequence was used to acquire high-resolution anatomical images with the following parameters: repetition time/echo time = 8.5 ms/3.2 ms, flip angle = 15°, voxel size = 1 × 1 × 1 mm^3^, and 188 slices. Ten-min fMRI and PET were acquired simultaneously. fMRI data were collected using a single-shot echo-planar imaging sequence: repetition time/echo time = 2000 ms/30 ms, flip angle = 90°, gap = 0.8 mm, voxel size = 3.59 × 3.59 × 4.40 mm^3^, 33 slices covering the whole brain and 300 volumes with interleaved slice acquisitions. Both T1 and echo-planar imaging were generated with B0 calibration. PET emission data were  acquired with 3D list mode. An 18-s 2-point Dixon scan was acquired for MRI-based PET attenuation correction. The decay- and attenuation-corrected PET images were reconstructed using an ordered subset expectation maximization algorithm (8 iterations, 32 subsets, and full width at half maximum of a Gaussian filter of 3.0 mm) with ToF and point-spread function. The reconstructed PET image matrix was 192 × 192, the field of view was 350 × 350 mm^2^, and the voxel size was 1.82 × 1.82 × 2.78 mm^3^, with spatial resolution of 4.5 mm [25–27].

### ^18^F-FDG PET partial volume correction and quantification

^18^F-FDG PET image spatial normalization and ROI ^18^F-FDG SUVR calculation were described in our previous studies [28–30]. Briefly, all PET images were co-registered to matched T1-weighted 3D BRAVO MRI images. The structural MRI images were normalized to standard Montréal Neurological Institute (MNI) space using statistical parametric mapping software (SPM12, Wellcome Department of Imaging Neuroscience, London, United Kingdom) and CAT12 toolbox (http://dbm.neuro.uni-jena.de/cat/) with MRI template (image volume, 121 × 145 × 121; voxel size, 1.5 × 1.5 × 1.5 mm^3^ in x, y, z). The transformation parameters determined by MRI spatial normalization were then applied to the co-registered PET images for PET spatial normalization. To minimize partial volume effects on PET measurements, an iterative reblurred Van Cittert iteration method was applied to 10-min ^18^F-FDG PET images for partial volume correction (PVC), where the PVC image *T*_*k* + 1_(*x*) at *k*th iteration for image *I*(*x*) at voxel *x* is updated by following equation.

$$ {\mathrm{T}}_{\mathrm{k}}+1\left(\mathrm{x}\right)={\mathrm{T}}_{\mathrm{k}}\left(\mathrm{x}\right)+\upalpha \left(\mathrm{h}\ast \left(\mathrm{I}-\mathrm{h}\ast {\mathrm{T}}_{\mathrm{k}}\right)\right)\left(\mathrm{x}\right) $$

where *T*_0_(*x*) = *I*(*x*), *h*(*x*) is a 3D Gaussian kernel of 4.5-mm FWHM spatial smoothing function, step length α = 1.5, and the iteration was stopped if relative percent change of PVC images < 1% [28, 30]. ^18^F-FDG SUVR images were calculated on the partial volume corrected PET images using cerebellum as reference tissue. The ROIs were then used to extract the SUVR on ^18^F-FDG SUVR images.

### Structural MRI analysis

The 3D high-resolution T1-weighted images data was processed using DPARSF voxel-based morphometry toolbox CAT12 based on SPM12 by adopting standard voxel-based morphometric (VBM) analysis processing routine. After normalizing all subjects to the standard MNI apace, segmenting (DARTEL) into gray matter, white matter, and cerebrospinal fluid images, the resulting images were modulated (without including affine component) and smoothed using a 4-mm FWHM 3D Gaussian filter. The gray matter volumes of total hippocampus and the hippocampal subregions were extracted based on the smoothed gray matter maps.

### Preprocessing of fMRI data

The SPM12 and Data Processing Assistant for Resting-State fMRI (DPARSF) toolbox (https://www.nitrc.org/projects/dparsf/) [31] were used to perform fMRI data preprocessing. The first 10 functional volumes were removed to stabilize the fMRI signal and adapt the participants to the circumstances. The remaining 290 volumes were realigned to the middle volume for the head motion correction and then registered to the T1-weighted sagittal images after slice timing correction. Nuisance regressors included the six motion vectors computed during the rigid body head motion correction. We regressed out 24 nuisance parameters (12 movement parameters and their first derivative). Eight participants (1 NC, 4 MCI, and 3 AD) were excluded based on excessive head motion (maximal translation > 3 mm or rotation > 3°). Then, the individual T1-weighted image was co-registered to the mean functional image using a linear transformation and segmented into gray matter, white matter and cerebrospinal fluid tissue probabilistic maps using the SPM12 unified segmentation algorithm. After normalizing to MNI standard template and smoothing with FWHM = 4 mm, the spatially normalized functional images were resampled to 3-mm isotropic voxels, and all voxel time courses were then temporally bandpass filtered with frequencies from 0.01 to 0.1 Hz to reduce the effect of low frequency drifts and high-frequency physiological noise.

### Seed-based functional connectivity analysis

**Fig. 1:**
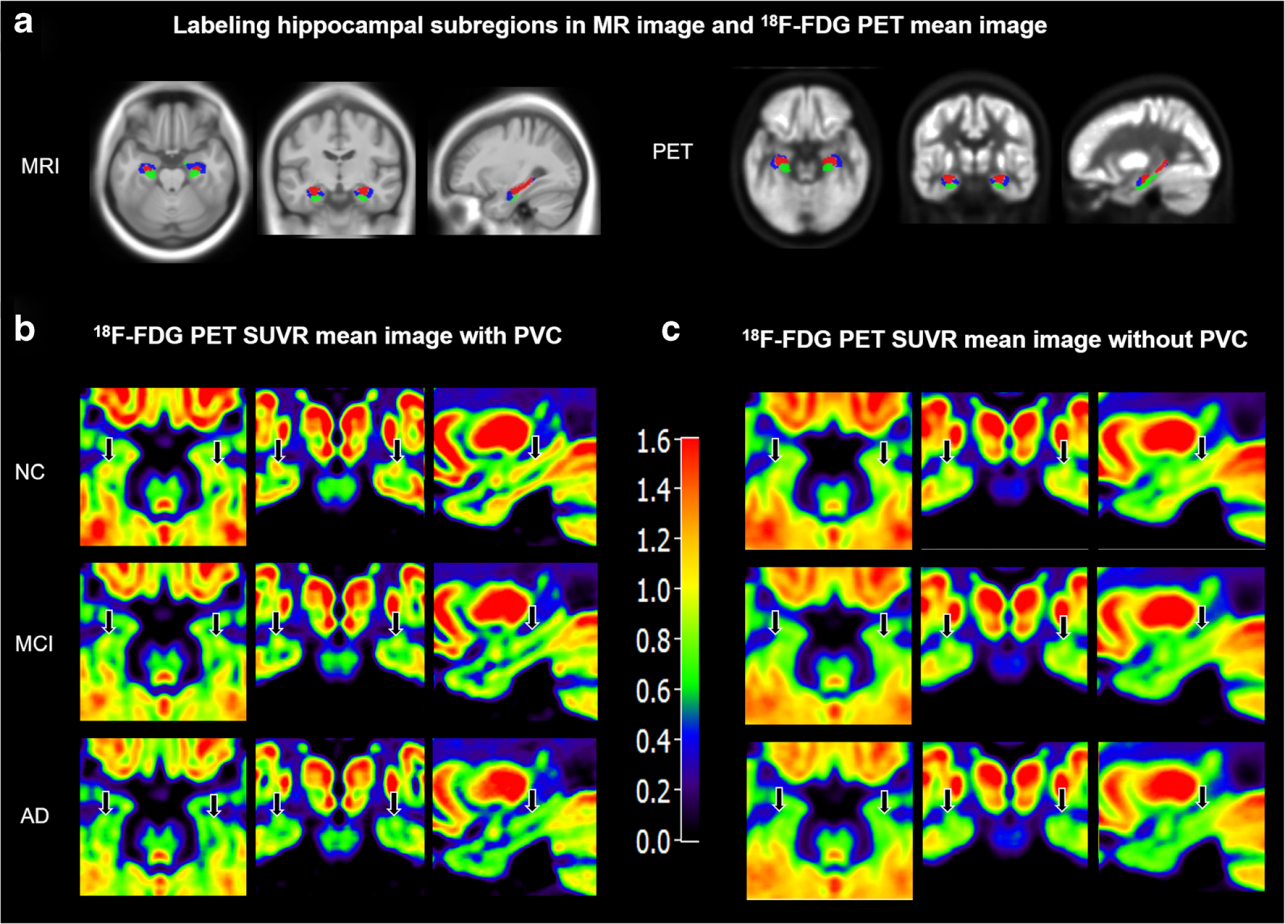
Hippocampal subregions masks and mean ^18^F-FDG PET SUVR images with and without PVC. Hippocampal subregions masks overlaid on template MRI and PET in standard MNI space. Blue, CA1; red, CA2/3/DG; and green, subiculum. The mean SUVR images with PVC (**b**) illustrate increased contrast and spatial resolution as compared to the mean SUVR images without PVC (**c**). Note that the mean images are averaged over all participants in each group. The black arrows indicate the location of the hippocampus

The masks of the region of interests (ROIs) (bilateral and unilateral hippocampus, CA1, CA2/3/DG, and subiculum) were extracted respectively from probabilistic cytoarchitectonic maps of the SPM Anatomy Toolbox for ROI-based functional connectivity analysis [32] (Fig. 1). After bandpass filtering all voxel time courses for frequencies from 0.01 to 0.1 Hz, the voxel time courses of the ROIs were extracted, and then the functional connectivity of the hippocampal subregions was calculated within DPARSF. The individual mean time courses were extracted for bilateral and unilateral hippocampus, CA1, CA2/3/DG, and subiculum. Then the individual ROI-based functional connectivity maps were computed for each region. Subsequently, we extracted and averaged the statistically significant ROI values from single-subject functional connectivity maps after Fisher’s z transformation.

### Statistical analysis

Statistical Analysis System (SAS version 9.4, SAS Institute, Inc.) was used for all statistical analyses. Gender was compared with a chi-squared test. Each group comparison analysis in connectivity maps (obtained from the bilateral and unilateral hippocampus, CA1, CA2/3/DG, and subiculum region, respectively) was performed using two-sample *t* tests. The significance levels of all clusters analyses were set to the voxel level at *P* < 0.01 and the cluster level at *P* < 0.05, after Gaussian random field correction. Age, gender, and education were used as covariates. A generalized linear model was used for group comparison on ROI volume and ^18^F-FDG SUVR. A partial Pearson’s correlations between ROIs connectivity, ^18^F-FDG SUVR, and clinical assessments were calculated in all groups.

## Results

### Demographics

A total of 42 NC, 38 MCI, and 22 AD participants were studied. The demographic characteristics of all participants are listed in Table 1. Significant group differences were found in mini mental status examination (MMSE) scores, but not found (*P* > 0.05 for all) in age, gender, and education. The AD (*P* < 0.001) and MCI (*P* < 0.001) group had significantly lower MMSE scores compared with NC.

**Table 1:** Demographics and neuropsychological assessments

Parameter	NC	MCI	AD	*F* value	*P* value
Total no. of participants	42	38	22	–	–
Women	18	20	13	1.69	0.430
Mean age in years (SD)	65.81 (6.14)	68.89 (8.46)	67.68 (7.17)	1.81	0.169
Mean education in years (SD)	12.98 (2.61)	12.34 (3.38)	11.95 (3.12)	0.93	0.399
Mean MMSE (SD)	28.83 (1.4)	26.00 (2.98)	19.64 (6.19)	49.94	< 0.001
CDR	0	0.5	0.5–1	–	–

Group comparisons: generalized linear model (age, education, MMSE), gender (chi-square test)

### ^18^F-FDG PET hypometabolism in hippocampal subregions

**Fig. 2:**
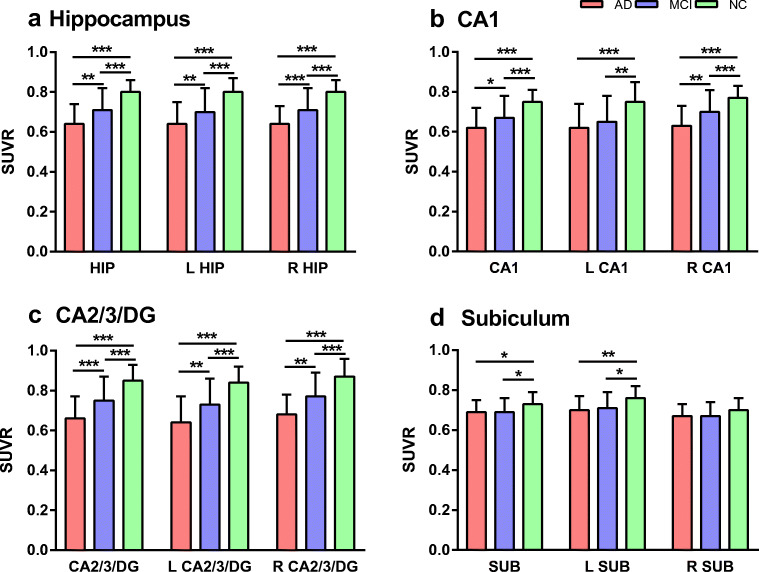
Group differences in ^18^F-FDG SUVR of hippocampus and hippocampal subregions. Bar graphs display the mean (standard deviation) of the age-, gender-, and education-corrected ^18^F-FDG SUVR. Mean ^18^F-FDG SUVR reductions in patients with AD and MCI compared with (*P* < 0.05) NC in hippocampus (**a**), CA1 (**b**), CA2/3/DG (**c**), and subiculum (**d**). Red, AD; blue, MCI; and green, NC. *P* value as defined using a generalized linear model to detect significant difference in the three groups. *P < 0.05, **P < 0.01, ***P < 0.001

The ^18^F-FDG SUVR mean images of hippocampus in Fig. 1 showed that the PVC remarkably improved spatial resolution and contrast. Therefore, the results here after presented in the study focused on the partial volume corrected SUVRs. Patient groups have significantly decreased ^18^F-FDG PET metabolism compared to NC. In patients with AD, significant reduction of ^18^F-FDG SUVR was observed within hippocampus, left hippocampus, right hippocampus, bilateral CA1, left CA1, right CA1, bilateral CA2/3/DG, left CA2/3/DG, right CA2/3/DG, bilateral subiculum, and left subiculum regions compared with those of NC. ^18^F-FDG SUVR within hippocampus, left hippocampus, right hippocampus, right CA1, bilateral CA2/3/DG, and left CA2/3/DG regions was significantly reduced in the AD patients compared with those of MCI. Compared with NC, MCI patients ^18^F-FDG SUVR was significantly reduced within hippocampus, left hippocampus, right hippocampus, right CA1, bilateral CA2/3/DG, left CA2/3/DG, right CA2/3/DG, bilateral subiculum, and left subiculum regions. However, only minor difference between AD and MCI patients was found in the left CA1, subiculum, left subiculum, and right subiculum regions (Fig. 2, Table 2).

**Table 2:** Averaged ^18^F-FDG SUVR in hippocampal subregions

Regions	AD	MCI	NC	*F* value	*P* value
Hippocampus	0.64 ± 0.10^bc^	0.71 ± 0.11^b^	0.80 ± 0.06	24.59	< 0.001
L Hippocampus	0.64 ± 0.11^bd^	0.70 ± 0.12^b^	0.80 ± 0.07	19.33	< 0.001
R Hippocampus	0.64 ± 0.09^bd^	0.71 ± 0.11^b^	0.79 ± 0.06	18.49	< 0.001
CA1	0.62 ± 0.10^bc^	0.67 ± 0.11^d^	0.75 ± 0.06	16.45	< 0.001
L CA1	0.62 ± 0.12^b^	0.65 ± 0.13^b^	0.75 ± 0.10	13.13	< 0.001
R CA1	0.63 ± 0.10^bc^	0.70 ± 0.11^b^	0.77 ± 0.06	16.24	< 0.001
CA2/3/DG	0.66 ± 0.11^bd^	0.75 ± 0.12^b^	0.85 ± 0.08	22.90	< 0.001
L CA2/3/DG	0.64 ± 0.13^bd^	0.73 ± 0.13^b^	0.84 ± 0.08	21.72	< 0.001
R CA2/3/DG	0.68 ± 0.10^bd^	0.77 ± 0.12^b^	0.87 ± 0.09	19.28	< 0.001
Subiculum	0.69 ± 0.06^b^	0.69 ± 0.07^a^	0.73 ± 0.06	6.11	0.003
L Subiculum	0.70 ± 0.07^b^	0.71 ± 0.08^b^	0.76 ± 0.06	7.69	0.001
R Subiculum	0.67 ± 0.06^a^	0.67 ± 0.07^a^	0.70 ± 0.06	3.95	0.022

^a^compared with NC, *P* < 0.05

^b^compared with NC, *P* < 0.01

^c^compared with MCI, *P* < 0.05

^d^compared with MCI, *P* < 0.01

### Functional connectivity in hippocampal subregions

**Fig. 3:**
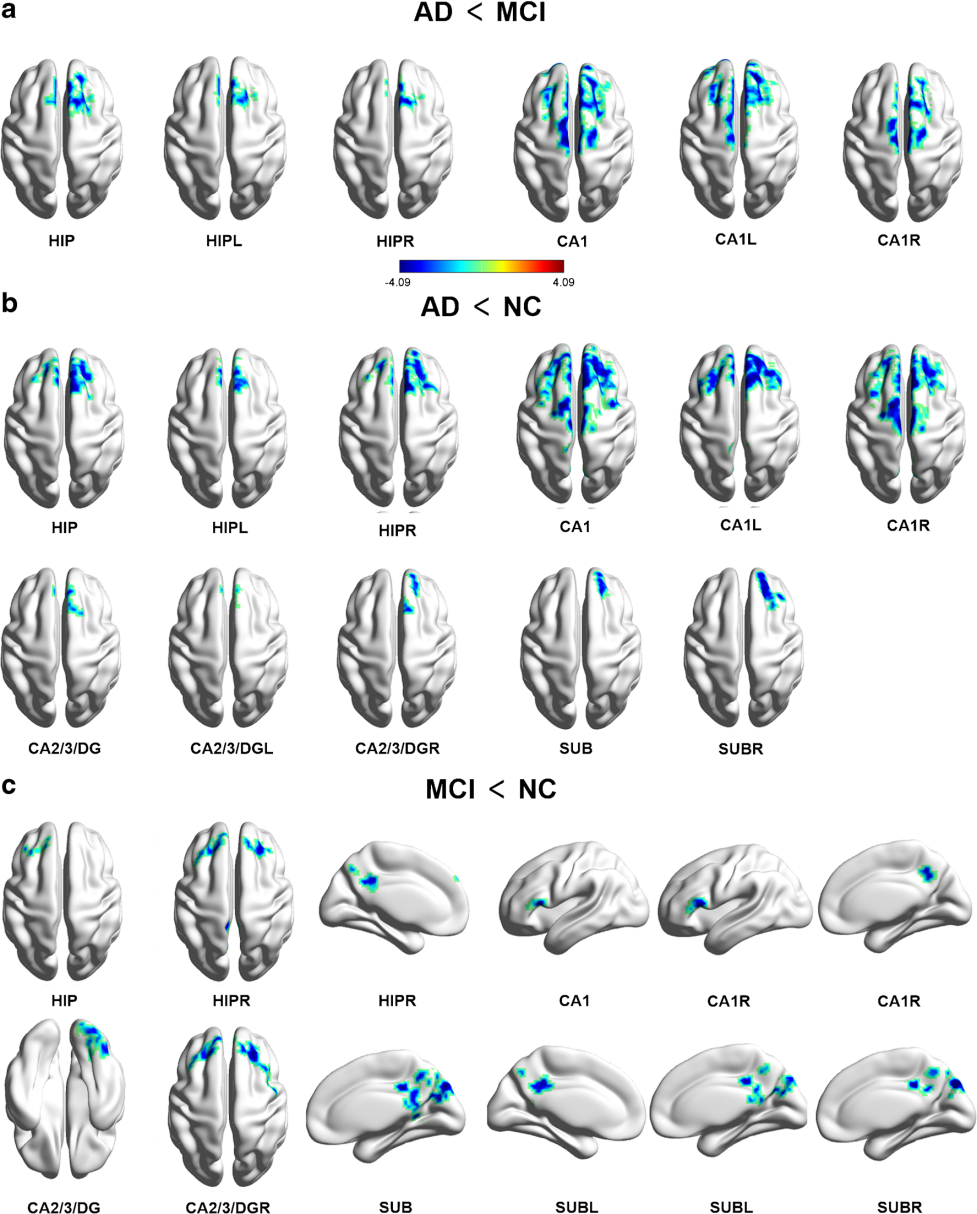
Group differences in hippocampal subregional functional connectivity. The results were mapped on the brain surface using BrainNet Viewer [48] showing reduced hippocampal subregional functional connectivity in AD compared with MCI (**a**) or NC (**b**), MCI compared with NC (**c**) (two-sample *t* test, voxel level *P* < 0.01, cluster level *P* < 0.05, Gaussian random field corrected). Colors indicate t scores

Group differences between NC and patient groups in functional connectivity were illustrated in Fig. 3 and Table 3. In AD patients, a significant decrease (*P* < 0.05) in functional connectivity, in comparison with MCI patients, was found between the following pairs of regions: (1) hippocampus and both the right superior frontal gyrus (SFG) and bilateral superior medial frontal gyrus (SMFG); (2) left hippocampus and both right SFG and bilateral SMFG; (3) right hippocampus and bilateral SMFG; (4) bilateral CA1 and all right SFG, bilateral SMFG, supplementary motor cortex (SMC), and middle frontal gyrus (MFG); (5) left CA1 and all bilateral SFG, SMFG, SMC, and right MFG; (6) right CA1 and all bilateral SFG, SMC, and SMFG. No difference was found within the CA2/3/DG and subiculum regions (Fig. 3a).

**Table 3:** Group differences in subregional hippocampal functional connectivity

Seed regions	Cluster	Cluster size	*T* value	MNI (x, y, z)
AD < MCI				
Hippocampus	R SFG & L/R SMFG	273	− 4.512	15, 18, 60
L Hippocampus	R SFG & L/R SMFG	169	− 4.087	12, 21, 57
R Hippocampus	L/R SMFG	152	− 4.523	6, 36, 54
CA1	L/R SFG, SMFG, SMC & MFG	1190	− 4.958	− 6, − 15, 72
L CA1	L/R SFG, SMFG, SMC & R MFG	958	− 4.847	− 6, 30, 51
R CA1	L/R SFG, SMC & SMFG	339	− 4.516	6, 36, 54
AD < NC				
Hippocampus	L/R SMFG & SFG	476	− 4.759	6, 36, 57
L Hippocampus	L/R SMFG	209	− 4.396	6, 42, 51
R Hippocampus	L/R SMFG, SFG & R MFG	488	− 5.069	9, 27, 60
CA1	L/R SMFG, SFG, MFG & SMC	1286	− 5.620	6, 27, 57
	L/R Precuneus	152	− 4.178	− 3, − 57, 72
L CA1	L/R SMFG, SFG	987	− 5.069	6, 27, 57
	L/R Precuneus	131	− 4.534	− 6, − 57, 72
R CA1	L/R SMFG, SFG & MFG	820	− 5.412	6, 27, 57
	L/R Precuneus	115	− 3.949	0, − 69, 51
CA2/3/DG	L/R SMFG	109	− 4.190	6, 36, 57
L CA2/3/DG	R SMFG	62	− 3.497	9, 42, 51
R CA2/3/DG	R SFG	50	− 3.495	15, 42, 39
Subiculum	R SFG	57	− 3.821	21, 42, 39
R Subiculum	R SFG	153	− 4.205	21, 51, 33
MCI < NC				
Hippocampus	L MFG & SFG	64	− 3.701	− 36, 36, 39
R Hippocampus	L Precuneus	93	− 3.533	− 9, − 42, 33
CA1	L IFG	71	− 3.959	− 36, 3, 18
R CA1	L IFG	64	− 3.832	− 48, 27, 9
	R Precuneus	75	− 3.547	12, − 60, 39
CA2/3/DG	R Inferior Occipital gyrus & Lingual	151	− 3.695	21, − 87, − 6
R CA2/3/DG	L/R SFG & L/R MFG	169	− 4.406	− 27, 42, 39
Subiculum	L/R Middle Cingulate & PCC	424	− 4.335	6, − 36, 36
	R MOG, Superior Occipital gyrus & Cuneus	489	− 4.654	9, − 84, 39
L Subiculum	L/R Middle Cingulate & Precuneus	249	− 4.175	6, − 36, 36
	R MOG, Angular & Cuneus	311	− 4.231	39, − 72, 27
R Subiculum	R MOG & Cuneus	225	− 5.256	12, − 81, 39
	L/R Precuneus	173	− 4.583	6, − 36, 36

Voxel level *P* < 0.01 and the cluster level *P* < 0.05, after Gaussian random field correction

Compared with NC, AD patients showed a significant decrease in functional connectivity between the following pairs of regions: (1) hippocampus and bilateral SMFG and SFG; (2) left hippocampus and bilateral SMFG; (3) right hippocampus and all bilateral SMFG, SFG, and right MFG; (4) bilateral CA1 and all bilateral SMFG, SFG, MFG, SMC, and precuneus; (5) left CA1 and all bilateral SMFG, MFG, and precuneus; (6) right CA1 and all bilateral SMFG, SFG, MFG, and precuneus; (7) bilateral CA2/3/DG and all bilateral SMFG, left CA2/3/DG, and right SMFG; (8) right CA2/3/DG and right SFG; (9) bilateral subiculum and right SFG; and (10) right subiculum and SFG (*P* < 0.05). No difference was found within the left subiculum region (Fig. 3b).

In MCI patients, a significant decrease in functional connectivity, in comparison with NC (*P* < 0.05), was found between the following pairs of regions: (1) hippocampus and both left MFG and SFG; (2) right hippocampus and left precuneus; (3) bilateral CA1 and left inferior frontal gyrus (IFG); (4) right CA1 and both left IFG and right precuneus; (5) CA2/3/DG and both right inferior occipital gyrus and lingual; (6) right CA2/3/DG and all left SFG, right SFG, and bilateral MFG; (7) subiculum and all bilateral middle cingulate cortex, PCC, middle occipital gyrus (MOG), superior occipital gyrus, and cuneus; (8) left subiculum and all bilateral middle cingulate, precuneus, right MOG, angular, and cuneus; and (9) right subiculum and all right MOG, cuneus, and bilateral precuneus. No difference existed within the left hippocampus, CA1, and CA2/3/DG regions (Fig. 3c).

### Association between functional connectivity and MMSE

**Fig. 4:**
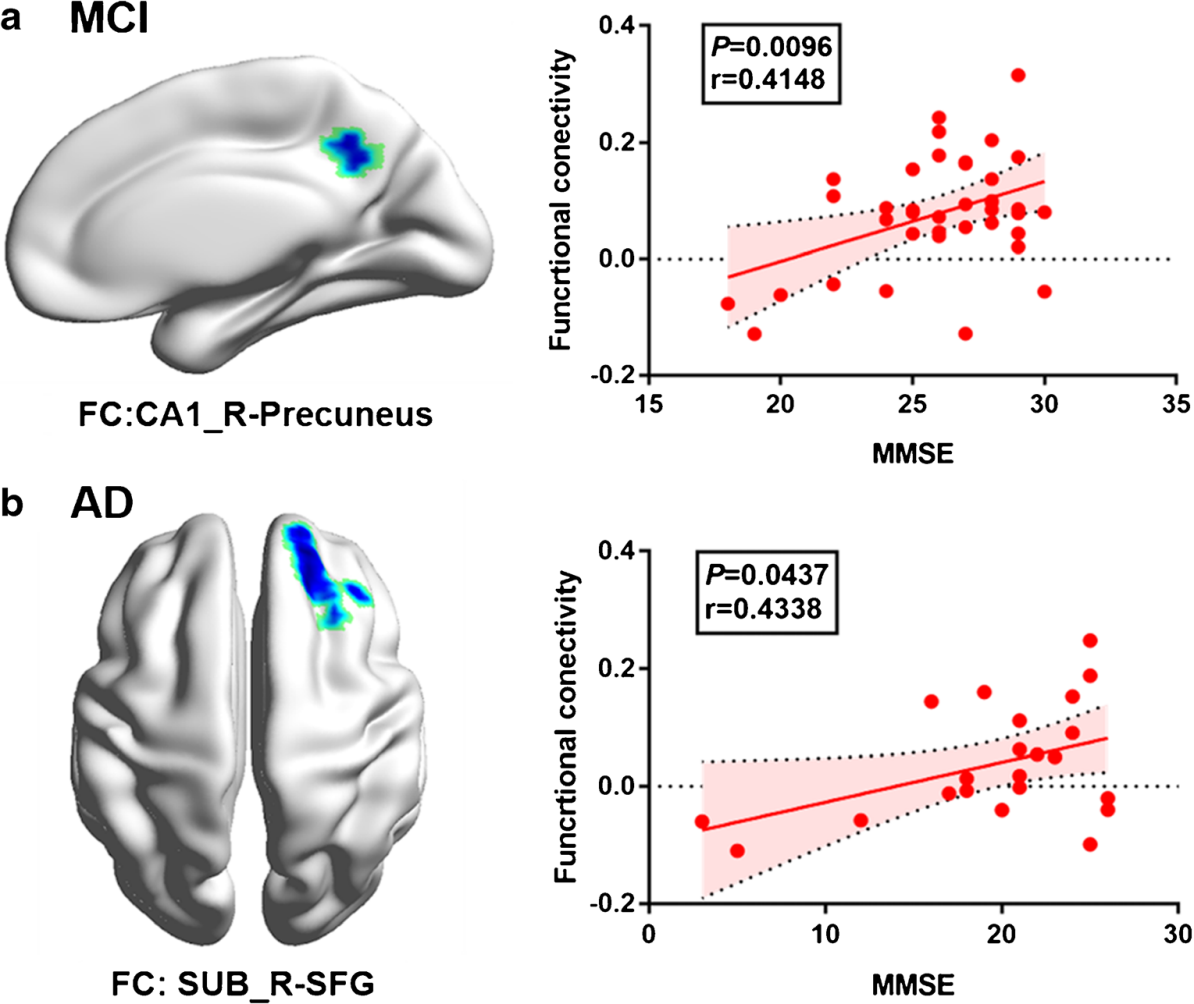
Correlations between hippocampal subregional functional connectivity and MMSE. **a** Decreased right CA1-precuneus functional connectivity in patients with MCI was positively correlated with MMSE. **b** Decreased right subiculum-superior frontal gyrus functional connectivity in patients with AD was positively correlated with MMSE. Fitted lines, *P* values, and 95% confidence intervals are displayed from linear regression models

The MMSE score predicted a higher right CA1 region functional connectivity with the right precuneus in patients with MCI (*P* = 0.010, *r* = 0.415; Fig. 4a) while a higher right subiculum region functional connectivity with the right SFG in patients with AD (*P* = 0.044, *r* = 0.434; Fig. 4b). There was neither association between MMSE and other hippocampal subregions in functional connectivity nor between MMSE and all the hippocampal subregions in ^18^F-FDG SUVR (all *P >* 0.05).

### Association among functional connectivity, ^18^F-FDG SUVR, and volume in hippocampal subregions

**Fig. 5:**
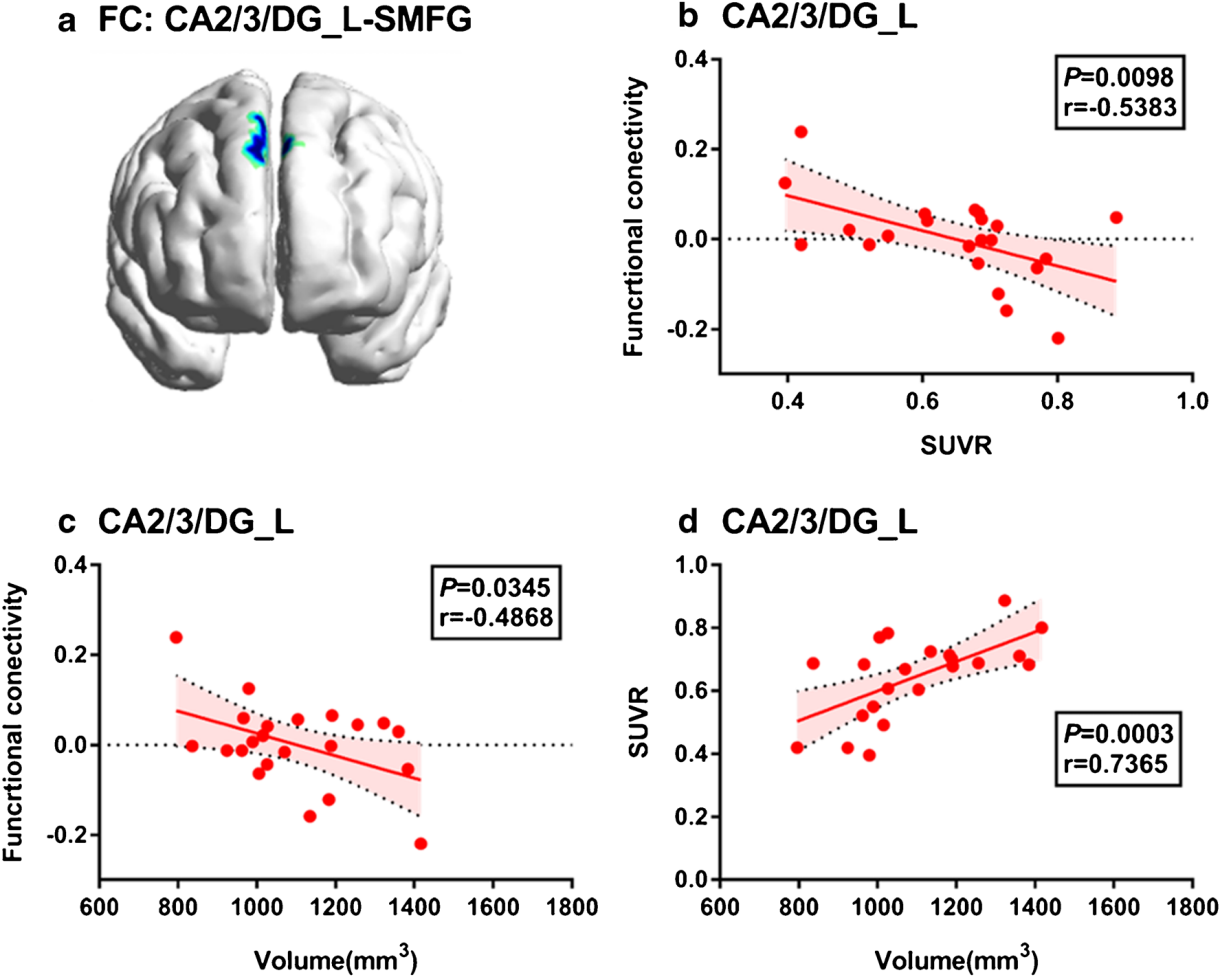
Association among left CA2/3/DG region functional connectivity, ^18^F-FDG SUVR, and volume in AD subjects. Decreased left CA2/3DG-SMFG connectivity in AD compared to NC group (**a**) was associated with local ^18^F-FDG SUVR (**b**) and volume (**c**). The left CA2/3DG volume in AD was associated with local ^18^F-FDG SUVR (**d**). Fitted lines, *P* values, and 95% confidence intervals are displayed from linear regression models

**Fig. 6:**
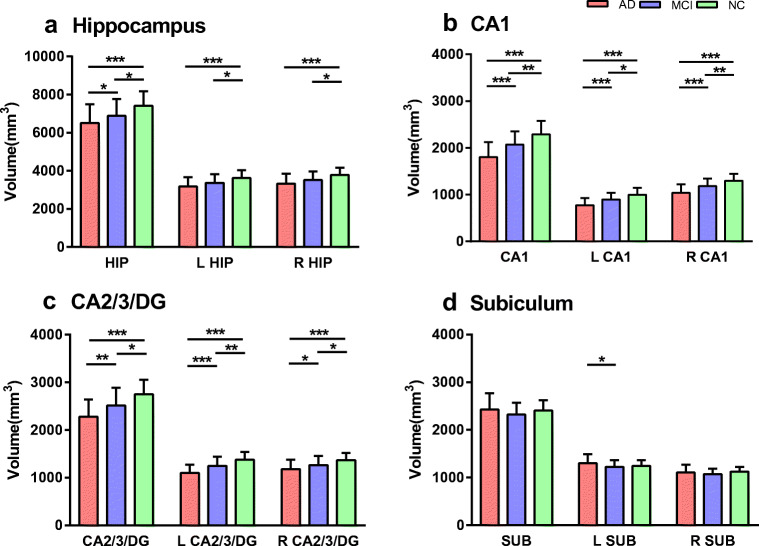
Group differences in volumes of hippocampus and hippocampal subregions (mean ± standard deviation, mm^3^). Generalized linear model followed by least square means post hoc analysis showed that the volume reduced in patients with AD and MCI compared with NC in hippocampus (**a**), CA1 (**b**), CA2/3/DG (**c**), and subiculum (**d**) with the age-, gender-, and education-corrected (*P* < 0.05). Red, AD; blue, MCI; and green, NC. *P < 0.05, **P < 0.01, ***P < 0.001

Partial Pearson’s correlation analysis demonstrated that the decreased left CA2/3DG-SMFG connectivity in AD compared to NC group was associated with left CA2/3DG higher ^18^F-FDG SUVR (*P* = 0.010, *r* = −0.538; Fig. 5a, b) and volume (*P* = 0.035, *r* = − 0.487; Fig. 5c). There was a linear correlation between the left CA2/3DG volume and ^18^F-FDG SUVR in AD patients (*P* < 0.001, *r* = 0.737; Fig. 5d). No correlation was found between functional connectivity and metabolism in other hippocampal subregions (all *P >* 0.05). The volumes of the hippocampal subregions are shown in Fig. 6.

## Discussion

To the best of our knowledge, this is the first study to evaluate subregional hippocampal resting-state brain functional connectivity and glucose metabolism in AD study using simultaneous PET/fMRI. Hybrid PET/MRI is capable of simultaneous evaluating resting-state intrinsic activity, glucose metabolism, and gray matter volume, which could provide evidence for better understanding the neurodegenerative mechanisms underlying AD. Our hippocampal subregion-based analysis on simultaneous ^18^F-FDG PET/fMRI demonstrated that patient groups had significantly reduced functional connectivity and ^18^F-FDG SUVR in comparison with NC in most of the hippocampal subregions. Specifically, we found a negative correlation between the decreased left CA2/3DG-SMFG connectivity and local ^18^F-FDG PET hypometabolism in AD patients. In addition, we observed that the right CA1-precuneus connectivity was associated with cognitive impairment in participants with MCI.

Our inter-group subregion-based analysis on fMRI showed that all hippocampal subregions in both patient groups (AD and MCI) had decreased functional connectivity comparing to NC, and CA1 subregion could potentially serve as a major imaging indicator differentiating AD and MCI patients. A recently published study analyzed specific functional connectivity of CA1, subiculum, and CA2/3/4/DG using healthy elderly and their changes in patients with amnestic MCI [12]. The authors found a significant reduced connectivity within the subiculum network (with frontal cortex and PCC) in amnestic MCI patients. Our current findings are partly in line with this result, showing that the individuals with MCI had reduced connectivity between subiculum and PCC, right MOG, superior occipital gyrus, and cuneus. Besides, our study also demonstrated that the hippocampus, CA1, and CA2/3/DG had reduced connectivity in patient groups compared with NC, mainly in the frontal cortex including SMFG, SFG, precuneus, PCC, etc. Using the whole hippocampus as a seed, we showed that the right whole hippocampus-medial prefrontal cortex, cingulate cortex, right cuneus extending into precuneus, left cuneus, and PCC functional connectivity were disrupted [18]. More specifically, as shown in Fig. 3a, CA1 was the only subregion exhibiting group differences between AD and MCI patients. The functional connectivity of CA1 decreased in patients with AD mainly in the frontal cortex and precuneus areas, which are the hubs of the default mode network. These results concurred well with a neuroimaging study that showed reduced hippocampal functional connectivity in AD [33] and also confirmed our earlier study of resting-state fMRI functional connectivity using the total hippocampus as a seed [34].

Our hippocampal subregional analysis on ^18^F-FDG PET metabolism showed that both patient groups possessed reduced SUVR in hippocampal subregions (AD < MCI < NC), and the decreased left CA2/3/DG-SMFG connectivity in AD compared to NC group exhibited a negative correlation with the left CA2/3/DG ^18^F-FDG PET metabolism in AD patients. A recent study of Choi et al. focused on comparing the glucose metabolism of hippocampal subregions in mild-AD patients and healthy controls [15]. Their results revealed that the reductions in metabolic activity were found varying along the hippocampal axis in early-stage AD patients. When considering the hippocampal body as an entire structure, there was significantly lower glucose metabolism in the AD group than that of control group just in the left CA2/3 and CA4/DG. Apart from the subregions specified by Choi et al., our results showed that all of the hippocampal subregions had reduced metabolism except for the right subiculum, which was supported by our volume results. The subiculum gray matter volume reduction in participants with MCI and AD is not consistent in the previous studies [35–37]. Although we found a trend of subiculum gray matter volume reduction in participants with MCI and AD as compared to healthy controls (*P* = 0.5 and *P* = 0.1, respectively) which is consistent with previous amnestic MCI study [37], the reduction of subiculum volume still required further investigation and validation. The novelty of our current study is the relationship between metabolism and strength of hippocampal subregions functional connectivity to remote brain regions in AD patients, which supports the hippocampus disconnection hypothesis, i.e., uncoupling of hippocampus from cortical inputs system may contribute to disinhibition like changes of intrahippocampal activity [38, 39]. As shown in Fig. 5b, the association between left CA2/3/DG-SMFG connectivity and ^18^F-FDG PET metabolism revealed a negative correlation and was specific for AD patients, without the presence in neither MCI patients nor NC participants. Contrary to a previous finding suggesting no relationship between functional connectivity and ^18^F-FDG PET metabolism in the posterior default mode network in the AD group [40], our results supported a recent evidence from a task fMRI study in humans indicating that greater hippocampal activation in amnestic MCI localized in the CA3/DG region [37], suggesting similar neural dysfunction. A therapeutic study in amnestic MCI showed that levetiracetam could reduce CA3/DG activation and improve cognitive function [41], indicating that targeting excess hippocampal activity has therapeutic potential. Overall, by integrating ^18^F-FDG PET and fMRI data, we found a robust relationship between left CA2/3/DG-SMFG connectivity and local ^18^F-FDG SUVR in AD patients.

As for the correlation analysis of hippocampal subregions and MMSE, we found that the right CA1-precuneus and right subiculum-SMFG connectivity were positively correlated with cognitive impairment in MCI and AD patients, respectively. A previous animal research study showing that the pattern of CA1 neuron activation indicated cognitive features [42]. In patients with AD, the CA1 region was severely affected by neuron number and neurofibrillary tangle, which were significantly related to CDR scores [43]. A recent autopsy study also found that the degree of Lewy body pathology in CA1, but not CA2, predicted pre-mortem episodic memory impairment in patients with Lewy body dementia and Parkinson’s disease [5, 44]. Their findings suggested that CA1 might be more functionally relevant than CA2 and subiculum regions in memory impairment. These results supported the view that excess hippocampal activation directly contributed to the cognitive decline in prodromal AD [41, 45].

The partial volume effects on the subregional hippocampal neurodegeneration measurements by PET were minimized by voxelwise PVC. As demonstrated by Fig. 1 and Table 4, the statistical significance levels were improved by PET with PVC in testing the ROI FDG SUVR differences between AD and MCI groups in left and right hippocampus, CA2/3/DG, and right CA1. This is consistent with our previous Alzheimer’s Disease Neuroimaging Initiative studies in small ROIs including amygdala and entorhinal cortex, where the PET spatial resolution of 8-mm FWHM [28, 29]. In the study, we also performed RBV (region-based voxelwise)-RVC (reblurred Van Cittert iteration) PVC algorithm [46]. The statistical analysis based on PET with RBV-RVC PVC did not change any conclusions from RVC-based analysis (not shown). Note that the subregional hippocampal neurodegeneration measurements by ^18^F-FDG PET are consistent with the ones from high-resolution T1-weighted MRI (Figs. 2, 5, and 6). We also confirmed that the subregional hippocampal functional activities from the spatially smoothed fMRI (Gaussian 3D smooth filter with FWHM = 4 mm, see “Method” section) are separable with 3-T MRI scanners [16]. We realized that the biological AD definition was recently proposed by the National Institute on Aging and Alzheimer’s Association for the A (Aβ)-T (tau)-N (neuro-degeneration) research framework [47]. We will have AT(N) CSF or imaging measurements in future study to minimize AD pathology biases.

**Table 4:** Statistical significance of group difference in subregional hippocampal ^18^F-FDG SUVRs

Regions	AD vs MCI *P* value		AD vs NC *P* value		MCI vs NC *P* value	
	PVC	Non-PVC	PVC	Non-PVC	PVC	Non-PVC
Hippocampus	*0.002*	*0.010*	< 0.001	< 0.001	< 0.001	< 0.001
L Hippocampus	*0.010*	*0.023*	< 0.001	< 0.001	< 0.001	< 0.001
R Hippocampus	*< 0.001*	*0.008*	< 0.001	< 0.001	< 0.001	< 0.001
CA1	0.016	0.028	< 0.001	< 0.001	< 0.001	< 0.001
L CA1	0.171	0.110	< 0.001	< 0.001	0.002	< 0.001
R CA1	*0.002*	*0.011*	< 0.001	< 0.001	< 0.001	< 0.001
CA2/3/DG	*< 0.001*	*0.005*	< 0.001	< 0.001	< 0.001	< 0.001
L CA2/3/DG	0.003	0.008	< 0.001	< 0.001	< 0.001	< 0.001
R CA2/3/DG	*< 0.001*	*0.007*	< 0.001	< 0.001	< 0.001	< 0.001
Subiculum	0.598	0.345	0.018	0.002	0.032	0.010
L Subiculum	0.483	0.246	0.004	< 0.001	0.012	0.005
R Subiculum	0.971	0.575	0.1765	0.065	0.126	0.093

A generalized linear model including covariates age, gender, and education level was used to detect ^18^F-FDG ROI SUVR significant difference among NC, MCI, and AD groups. The *P* values were reduced (italic) for ROI SUVRs calculated from PET with PVC

In summary, the subregional hippocampal level analysis revealed hypometabolism, lower gray matter volume, aberrant functional connectivity, and their relationship in MCI and AD patients. In addition, the right CA1-precauneus connectivity was related to the cognition in MCI patients, and the left CA2/3/DG functional connectivity was correlated to hypometabolism in AD patients. Our findings demonstrate that the associations existed at subregional hippocampal level between the functional connectivity and neurodegeneration measured by simultaneous PET/MRI.
